# Does aerobic exercise associated with tryptophan supplementation attenuates hyperalgesia and inflammation in female rats with experimental fibromyalgia?

**DOI:** 10.1371/journal.pone.0211824

**Published:** 2019-02-20

**Authors:** Rafael Marins Rezende, Maria do Carmo Gouveia Pelúzio, Franciany de Jesus Silva, Emanuel Mattos Della Lucia, Lukiya Silva Campos Favarato, Hercia Stampini Duarte Martino, Antônio José Natali

**Affiliations:** 1 Department of Nutrition and Health, Federal University of Viçosa, Viçosa, MG-Brazil; 2 Department of Physical Education, Federal University of Viçosa, Viçosa, MG-Brazil; 3 Department of Veterinary Medicine, Federal University of Viçosa, Viçosa, MG-Brazil; Max Delbruck Centrum fur Molekulare Medizin Berlin Buch, GERMANY

## Abstract

The objective of this study was to verify the effects of aerobic exercise associated with tryptophan (TRP) supplementation on hyperalgesia, as well as on cortisol, IL-6 and TNF concentrations in female rats with experimental fibromyalgia (FM). Female Wistar rats (initial body weight: ~ 350 g; age: 12 months) were randomly divided into 5 groups: CON (Control); F (Fibromyalgia induced); FE (Fibromyalgia induced plus exercise); FES (Fibromyalgia induced plus exercise and TRP supplementation) and FS (Fibromyalgia induced plus TRP supplementation). Fibromyalgia was induced with two injections (20 μL) of acidic saline (pH 4.0) into the right gastrocnemius muscle with a 3-day interval. Control animals received the same doses of neutral saline (pH 7.4). The exercised animals underwent progressive low-intensity aerobic exercise (LIAE) on a treadmill (10–12 m/min, 30–45 min/day, 5 days/week) for three weeks. During this period, the supplemented animals received a TRP supplemented diet (210 g/week), while the others received a control diet. Mechanical hyperalgesia was evaluated weekly and serum cortisol and muscle IL-6 and TNF concentrations were assessed after three weeks of interventions. Experimental FM caused bilateral hind paw hyperalgesia and augmented serum cortisol and muscle IL-6 concentrations. After 3 weeks of interventions, LIAE alone reduced hyperalgesia (151%) and reduced serum cortisol concentrations (72%). Tryptophan supplementation itself diminished hyperalgesia (57%) and reduced serum cortisol concentrations (67%). Adding TRP supplementation to LIAE did not further reduce hyperalgesia significantly (11%), which was followed by an important decrease in muscle IL-6 concentrations (68%), though reduction in serum cortisol pulled back to 45%. Muscle TNF concentrations were not affected. In conclusion, the association of TRP supplementation to LIAE does not potentiate significantly the reduction of bilateral mechanical hyperalgesia promoted by LIAE in female rats with experimental FM, however an important decrease in IL-6 is evident.

## Introduction

Fibromyalgia (FM) is a syndrome characterized by chronic widespread pain, fatigue, anxiety and sleep, cognitive and mood disorders [1]. It is present in 2 to 5% of the general population, particularly in women between 50 and 80 years of age [2,3]. Fibromyalgia has been linked to neuroendocrine abnormalities, involving the main stress modulator system, the hypothalamic-pituitary-adrenal (HPA) axis, and "deficits" in endogenous pain modulation systems [4–6].

Women with FM exhibited high circulating levels of cortisol and noradrenaline as well as increased release of proinflammatory cytokines (i.e. IL-1β, TNFα, IL-6, IL-18) by monocytes [7, 8]. Thus, cytokines such as IL-6 and TNFα appear to be involved in the regulation of the HPA axis and the sympathetic nervous system related to the painful symptomatology in FM [9]. For example, intramuscular administration of IL-6 and TNFα induces musculoskeletal hyperalgesia in animals with chronic widespread experimental pain [10,11], which indicates that cytokines may play an important role in the pathogenesis of FM.

Non-pharmacological therapies have been recommenced for the management of FM. For example, exercise is strongly recommended as it promotes improvements in pain [12–15]. In his sense, low to moderate-intensity aerobic exercises are recommended [16] and moderate-intensity aerobic exercise is reported to reduce the systemic concentration of biomarkers indicative of stress (e.g., cortisol and noradrenaline) and inflammation (e.g. cytokines) in patients with FM [17]. Tryptophan (TRP) supplementation has also been used in the management of FM and other painful syndromes, inasmuch as low serum concentrations of TRP have been associated with conditions such as chronic fatigue syndrome and FM [18,19]. Tryptophan supplementation has been used in an attempt to increase its cerebral availability, which would potentiate serotonin actions [20, 21, 22]. Since serotonin is responsible for stimulating the HPA axis in response to stress [23], TRP would improve the function of cerebral serotonin and reduce the release of cortisol in stressful situations. Indeed, TRP supplementation has been shown to reduce cortisol release in stress-prone patients [24,25] and to benefit the treatment of pain in acute and chronic pain syndromes [26, 27].

Nevertheless, the benefits of the association of aerobic exercise with TRP supplementation in the management of FM are not known. Therefore, this study was designed to verify the effects of aerobic exercise associated with TRP supplementation on hyperalgesia, as well as on cortisol, IL-6 and TNF concentrations in female rats with experimental FM.

## Materials and methods

### Animals and experimental procedures

The experimental procedures were approved by the Ethics Committee for Animal Use of the Federal University of Viçosa (process number 21/2015) and were conducted according to the Guide for the Care and Use of Laboratory Animals (2011), National Academy of Sciences (US).

Twelve-month old female adult *Wistar* rats (initial body weight: ~ 350 g) from the Central Biotherm of the Biological Center for Health Sciences at the Federal University of Viçosa (UFV) were randomly assigned to groups of 8 rats each: CON (Control); F (Fibromyalgia induced); FE (Fibromyalgia induced plus exercise); FES (Fibromyalgia induced plus exercise and TRP supplementation) and FS (Fibromyalgia induced plus TRP supplementation). During the experiment, animals were kept in individual cages in a temperature-controlled room (22 ± 2°C), with a light/dark cycle of 12/12 hours and had free access to water and diet.

After FM induction, the rats of the exercised groups were submitted to three weeks of low-intensity aerobic exercise, while those of the supplemented groups received a TRP supplemented diet. The body weight of the animals was measured once a week (on Fridays) during the experiment.

### Fibromyalgia induction

Repeated intramuscular injections of acidic saline are known to mimic the conditions of chronic and widespread pain [28]. The first injection produces transient hyperalgesia that decreases after 24h, and the second, administered 3 days later, promotes bilateral hyperalgesia for more than 4 weeks [28, 29]. Thus, the rats from F, FE, FES and FS groups received two unilateral intramuscular injections containing 20 μL of acidic saline (pH 4.0) in the right gastrocnemius muscle, while animals from CON groups received injections containing 20 μL of neutral saline (pH 7.4), with a 3-day interval between injections. For these fibromyalgia induction procedures, the animals were maintained under surgical sedation with isoflurane, sprayed with 100% FiO_2_, in an avalvular circuit and spontaneous breathing.

### Measurement of mechanical hyperalgesia

The mechanical hyperalgesia was measured following a protocol described previously [30]. In brief, three animals at a time were housed in individual boxes with wire-mesh floor and acrylic walls, which were placed on a raised platform. After 30 minutes of adaptation, a mechanical stimulus of increasing pressure (expressed in grams) was applied to the plantar surface of the right and left hind paws of each rat, using an electronic Von Frey apparatus (Insight, Ribeirão Preto—SP, Brazil). The mechanical stimulus was applied alternate 5 times to each hind paw of each animal in the pre-injection period and 3 times in the post-injection. Such stimuli were applied alternate to the left paw of each of the 3 rats and then to the left paw in the same order, so that each animal had and interval of approximately 30 seconds to receive the next stimulus to the contralateral hind paws. Pressure values were recorded by observing behaviors in nociceptive responses such as paw withdrawal, paw licking or jumping with all four legs. The withdrawal threshold in the pre-injection period was determined by calculating the median of the 5 measurements and then calculating how much each value deviated from the median. The 3 values that deviated less from the median were used to determine the mean and obtain the threshold value. The withdrawal thresholds in the post-injection period were determined by the mean of 3 consecutive pressure measurements. All measurements were taken in a quiet, temperature-controlled room at the same time of the day (8 to 10 a.m.), once a week (on Wednesdays). The same evaluator did all the tests blind for the treatments and the recorded pressure values. Mechanical hyperalgesia was measured before FM induction (pre-injection), 24 hours after the second injection of both acidic or neutral saline, and once a week for the following three weeks.

### Running velocity test and aerobic exercise training protocols

To determine the running speed (i.e. exercise intensity) during the exercise training sessions, the animals were submitted to a maximal running velocity (MRV) test on a treadmill (AVS, São Paulo-SP, Brazil), based on the total exercise time until fatigue (TTF) test, as previously described [31]. In summary, one week before the test the animals were adapted to the treadmill, on five consecutive days (5 min/day, 5% tilt up) and daily increases in treadmill velocity (8, 10, 11, 12 m/min). After the adaptation week, each animal performed three tests of progressive exercise until fatigue on three alternate days. During the tests, the initial velocity of the treadmill was 10 m/min (5% tilt up), being increased by 1 m/min every 3 minutes until fatigue which was defined when the animal could no longer keep the treadmill pace, when the test was interrupted. The mean MRV obtained by the animals in the TTF tests was calculated and established as a reference (i.e. 100% of MRV) to determine the exercise intensity during the exercise training sessions.

The aerobic exercise training was conducted on an electric-driven treadmill five times a week (Monday to Friday) for three consecutive weeks (Adapted from Sharma et al. [32]). The duration and intensity of exercise were gradually increased over the 3-week period. During the first week, the animals ran at 10 m/min (50% of the MRV), for 30 minutes. Then, on the second week, the animals ran at 11 m/min (55% of the MRV), for 40 minutes. And during the third week the running speed was 12 m/min (60% of the MRV), for 45 minutes. The exercise protocol included 2 minutes of warm-up (5 to 8 m/min) and 2 minutes of cooling down (5 to 8 m/min) within the total time of the running session. No incentive was used during the running.

### Diets and supplementation

The control and supplemented diets were purified and packed in pellets (RHOSTER, São Paulo, Brazil). The animals had free access to diet and water being the consumption *ad libitum* during the experimental period (3 weeks). Diets (210 g/week) were supplied twice a week (on Tuesdays’ and Thursdays’ in the morning). The animal home cage was cleaned every 2 days. On these days the leftovers in the feeder of each animal were separated and weighed to calculate weight gain, food consumption and food efficiency coefficient (FEC) individually.

The composition and profile of the amino acids of the diets are described in Table 1. The composition of the diets was based on the American Institute of Nutrition (AIN-93M) adult rodent maintenance diet [33] and adapted from the increase in concentration of TRP to the typical amino acid profile of casein (RHOSTER, São Paulo, Brazil).

**Table 1:** Composition and typical amino acid profile of control and experimental diets.

Nutrient	AIN-93M Diet	Experimental Diet
**Composition**	**g/Kg diet**	**g/Kg diet**
Casein (>85%protein)	140	140
Sucrose	100	100
Cornstarch	465,6	465,6
Dextrinized cornstarch	155	155
Fiber	50	50
Mineral mix	35	35
Vitamin mix	10	10
L-cystine	1,8	1,8
Choline bitartrate	2,5	2,5
Soybean oil	4	4
**Amino acid profile**	**g/Kg diet**	**g/Kg diet**
Isoleucine	9,0	8,2
Leucine	10,9	10,6
Phenylalanine	8,5	8,0
Tyrosine	7,4	7,5
Valine	10,5	9,9
Tryptophan	2,5	7,6
TRP/LNAA (%)	5,4	15,32

TRP/LNAA, the ratio of tryptophan to the sum of the other large neutral amino acids.

Reeves et al. (1993).

The typical amino acid profile estimated for the diets was analyzed by HPLC (CBO *Análises Laboratoriais*, Valinhos-SP, Brazil). The used TRP concentration was based on previous studies on protein supplementation enriched with alpha-lactalbumin, and its repercussion on plasma and brain concentrations of TRP and serotonin, respectively [20,21,22].

### Sample collection

At the end of the experimental period, 48 hours after the last exercise session, the animals were euthanized by decapitation in a clean room without strange noises. Euthanasia occurred on different days for different groups, but always occurred in the morning (8 am to 10 am). Immediately after decapitation, blood was collected by total exsanguination in separator gel tubes, which were subsequently centrifuged at 704 g (model Z216MK, Hermle, Germany) for 10 minutes. Serum was withdrawn and stored at -80°C for cortisol analyzes. The gastrocnemius muscle of the right limb was dissected, washed in cooled saline, frozen on dry ice and stored at -80°C for the quantification of IL-6 and TNF cytokine concentrations.

### Body weight and weight gain determination, food consumption, food efficiency coefficient (FEC)

The animals were weighed (Mettler Toledo, Brazil) once a week (Thursday’s mornings) during the experiment. The weight gain of each animal was calculated based on the equation: final weight—initial weight. The food consumption of each rat was evaluated weekly based on the amount of diet added minus the rest in the feeder, as described above. Food efficiency coefficient of each rat was calculated based on the ratio between animal weight gain and dietary intake.

### Determination of serum cortisol concentrations

Analysis of serum cortisol concentrations was performed in duplicate by ELISA (Enzyme-linked Immunosorbent Assay) using a commercial kit (Kit Cat. # EIA1887-CTS-Lot EIA 1887, MARBURG-German), as recommended by the manufacturer.

### Determination of muscle concentrations of IL-6 and TNF

The analyses of the muscle concentrations of IL-6 and TNF were performed using the multiplex immunoassay at the Specialized Laboratory in Scientific Analyzes (LEAC, São Paulo—Brazil). Muscle tissue (100 mg; gastrocnemius) was homogenized with 50 mM phosphate buffer (pH 7.0) and added with Tween-20 (0.05%) and aprotinin (5 mg/ml). The samples were then centrifuged at 8000 g (model Z216MK, Hermle, Germany) at 4°C for 5 minutes and the supernatants were removed. The xMAP (MAP = Multiple Analyte Profiling) technique was used, in which magnetic microspheres are stained with two different spectral fluorochromes. Each sphere has a "signature" based on "color code". Thus, the analyte binds, by means of non-reversible covalent bonds, to the capture antibodies located on the surface of the microspheres. Detection is done by means of a third fluorescent marker (phycoerythrin), bound to the detection antibody. The RECYTMAG-65K-02 kit (IL-6 and TNF, Millipore, USA) was used that uses these microspheres as the base of the multiplex immunoassay and doses all cytokines simultaneously. In this way, each bead is conjugated to a specific analyte antibody and read on the Luminex (manufacturer) equipment using a dual lasers system. A laser beam detects the microsphere (test specific color code) and the other laser quantifies the reporter signal in each microsphere. The minimum detection concentrations of this kit are 30.6 pg/mL for IL-6 and 1.9 pg/mL for TNF.

### Statistical analysis

Data were submitted to the Kolmogorov-Smirnov normality test. Data for body weight, weight gain, food consumption, FEC and cortisol, IL-6 and TNF concentrations were compared using one-way analysis of variance (ANOVA), followed by Tukey’s post-hoc test for multiple comparisons. Data for mechanical hyperalgesia were compared using two-way ANOVA repeated measures, followed Bonferroni’s post-hoc tests for multiple comparisons. Data were analyzed using SPSS (IBM SPSS Statistics for Windows, Version 19.0. Armonk, NY: IBM Corp) and a p value of < 0.05 was considered significant. Results are expressed as means ± standard deviation (SD).

## Results

### Body weight, weight gain, food consumption and food efficiency coefficient (FEC)

The initial body weight was not different between the experimental groups (Table 2). At the end of three weeks of interventions, body weight did not differ between groups. Likewise, the FEC was not different between the experimental groups.

**Table 2:** Body weight, weight gain, food consumption and coefficient of food efficacy.

Parameters	CON	F	FE	FES	FS	*p value*
**Initial weight** **(g)**	310,12 ± 12,96	298,25 ± 20,88	318,29 ± 19,72	293,14 ± 16,41	307,0 ± 20,17	>0,05
**Final weight** **(g)**	318,62 ± 17,18	310,75 ± 23,81	324,57 ± 24,50	298,28 ± 21,73	318,11± 24,63	>0,05
**Weight gain** **(g)**	8,5 ± 11,91	12,5 ± 11,75	6,28 ± 9,10	5,14 ± 14,78	11,11± 14,86	>0,05
**Food** **consumption** **(g)**	278,0 ± 42,44	279,75 ± 33,49	289,0 ± 47,51	236,66 ± 42,03	239,50± 40,31	>0,05
**CFE (%)**	2,90 ± 3,80	4,50 ± 3,90	1,90 ± 2,60	2,0 ± 6,40	3,11 ± 4,50	>0,05

FEC: food efficacy coefficient; CON, control; F, fibromyalgia induced; FE, fibromyalgia induced plus exercise; FES, fibromyalgia induced plus exercise and TRP supplementation; FS, fibromyalgia induced plus TRP supplementation. Data are means ± SD of 8 animals in each group. ANOVA one-way followed by Tukey.

### Mechanical hyperalgesia

**Fig 1:**
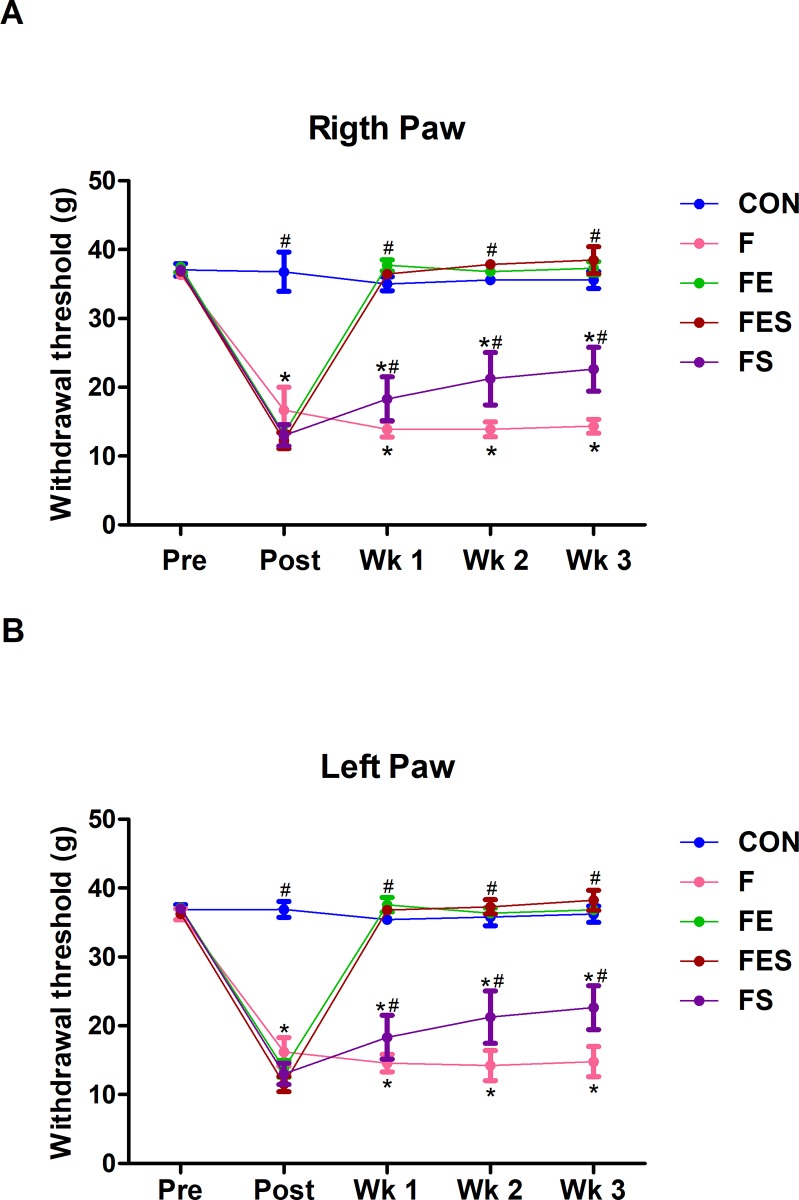
Mechanical hyperalgesia. (A) Withdrawal threshold of right paw. (B) Withdrawal threshold of left paw. CON, control; F, fibromyalgia induced; FE, fibromyalgia induced plus exercise; FES, fibromyalgia induced plus exercise and TRP supplementation; FS, fibromyalgia induced plus TRP supplementation; PRE, pre-injection; POST, post-injection. The data are means ± SD from 8 animals in each group. * p<0.05 vs CON group. ^#^ p<0.05 vs F group (ANOVA two-way repeated measures, followed by Bonferroni post hoc test).

Data presented Fig 1 refers to effects of interventions (i.e. aerobic exercise and supplementation) either alone or in combination on mechanical hyperalgesia at different moments. The withdrawal thresholds remained unchanged (p > 0.05) in both right and left paws in the CON group for all measurements (Fig 1A and Fig 1B), which indicates that neutral saline did not influence the mechanical sensitivity of these animals. However, at the post-injection moment the animals from F, FE, FES and FS groups exhibited decreased withdrawal threshold in both right and left paws (p <0.05), when compared to those from CON group, which demonstrates the effectiveness of acidic saline in inducing bilateral hyperalgesia.

Concerning the effects of interventions (Fig 1A and Fig 1B), exercise training (F vs FE) increased (p < 0.05) withdrawal threshold on both hind paws (i.e. reduced bilateral mechanical hyperalgesia) to control levels at weeks 1 (~ 162%), 2 (~ 157%) and 3 (~ 151%), while TRP supplementation (F vs FS) also reduced (p < 0.05) bilateral hyperalgesia to lesser extents at weeks 1 (~ 26%), 2 (~ 49%) and 3 (~ 67%). However, when treatments were combined (F vs FES) the bilateral hyperalgesia returned to control levels being the reductions respectively 156%, 166% and 162% at weeks 1, 2 and 3.

### Serum cortisol concentrations

**Fig 2:**
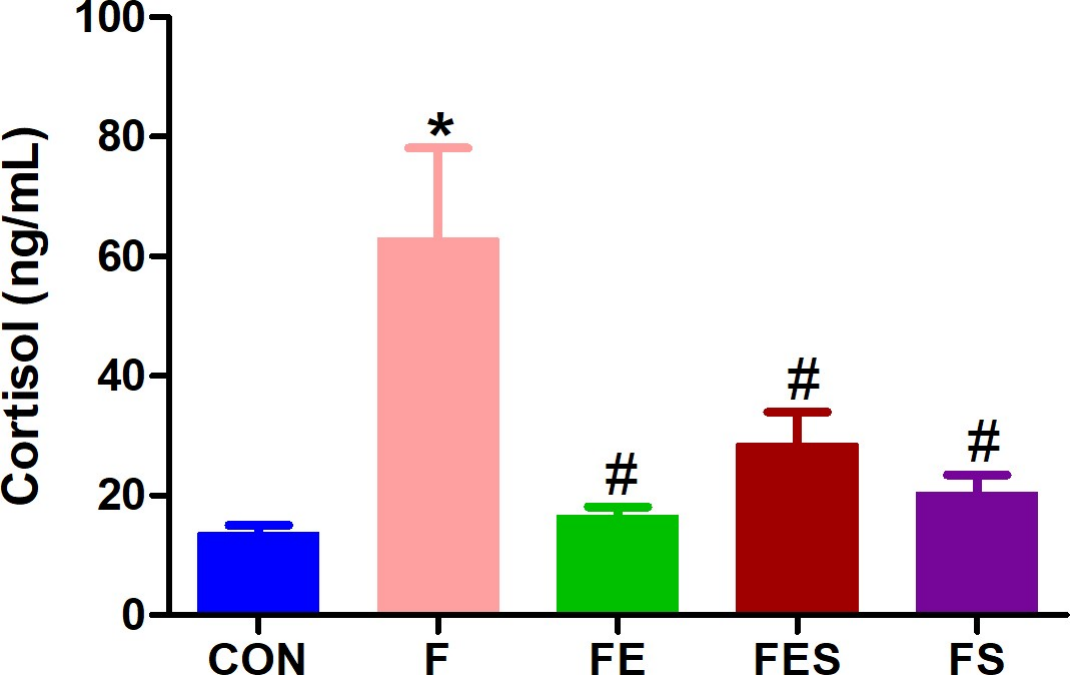
Serum cortisol concentrations. CON, control; F, fibromyalgia induced; FE, fibromyalgia induced plus exercise; FES, fibromyalgia induced plus exercise and TRP supplementation; FS, fibromyalgia induced plus TRP supplementation. Data are means ± SD from 6 to 8 animals in each group. * p<0.05 vs CON group. ^#^ p<0.05 vs F group (ANOVA one-way followed by Tukey’s post hoc test).

The results presented in Fig 2 refer to the cumulative effects of interventions (i.e. aerobic exercise and supplementation) either alone or in combination over three weeks on serum cortisol. Serum cortisol concentrations were higher (p <0.05) in animals from F group when compared to those from CON group, which indicates that the stress induced by acidic saline was sufficient to increase serum concentrations of serum cortisol in rats.

Regarding the effects of interventions, exercise training alone reduced (p <0.05) serum cortisol concentrations by 72% (F vs FE), while TRP supplementation itself reduced (p <0.05) it by 67% (F vs FS). The combination of treatments, however, reduced (p <0.05) it by 54% (F vs FES) only. This indicates that both interventions either alone or in combination play a role in reducing serum cortisol concentrations in this model.

### Muscle concentrations of IL-6 and TNF

**Fig 3:**
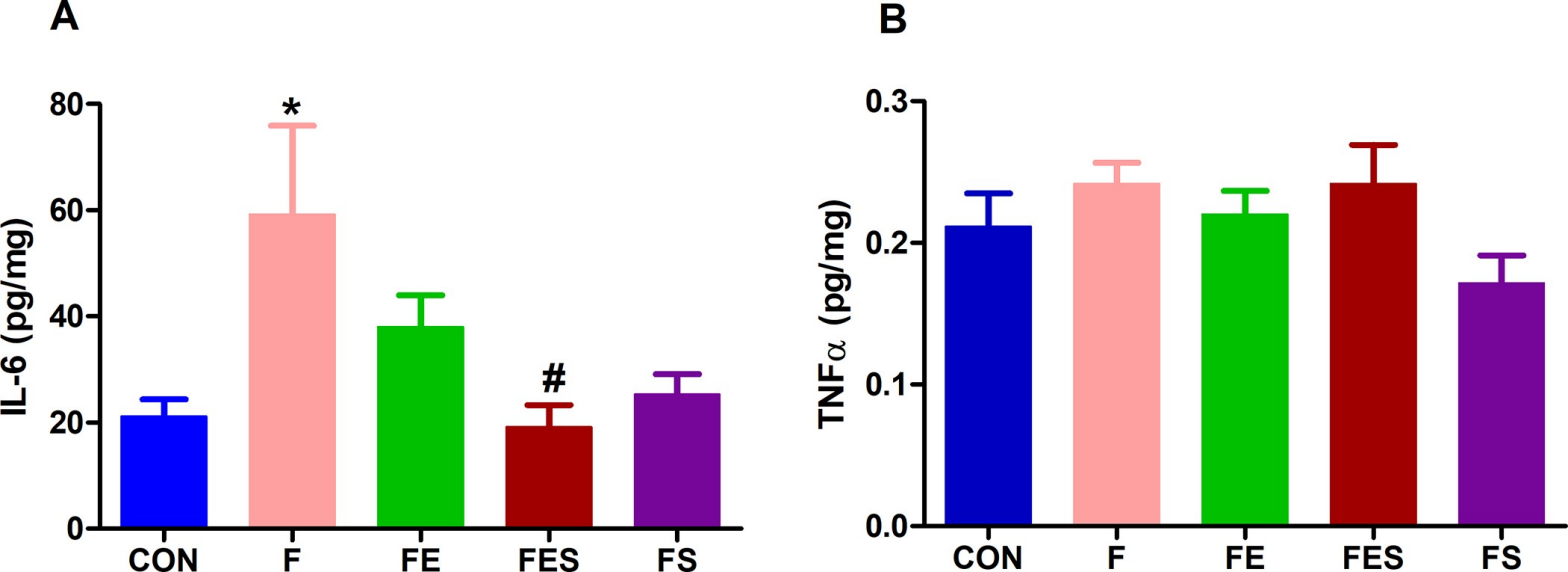
Concentrations of inflammatory cytokines in muscle tissue. (A) Interleukin-6 (IL-6). (B) Tumor necrosis factor (TNF). CON, control; F, fibromyalgia induced; FE, fibromyalgia induced plus exercise; FES, fibromyalgia induced plus exercise and TRP supplementation; FS, fibromyalgia induced plus TRP supplementation. Data are means ± SD from 6 to 8 animals in each group). * p<0.05 vs CON group. ^#^ p<0.05 vs F group (ANOVA one-way followed by Tukey’s post hoc test).

The results presented in Fig 3 also refer to the cumulative effects of interventions (i.e. aerobic exercise and supplementation) either alone or in combination over three weeks on muscle cytokines. Animals from F group showed higher (p <0.05) muscle IL-6 concentrations than those from CON group (Fig 3A). This indicates that neutral saline did not affect the muscle concentrations of this cytokine, whereas acidic saline promoted the increase in IL-6 concentrations.

Respecting the effects of interventions, the isolated effects of exercise training (F vs FE) and TRP supplementation (F vs FS) were not sufficient to reduce (p > 0.05) the muscle concentrations of IL-6. The combination of treatments (F vs FES), nevertheless, reduced (p < 0.05) the muscle concentrations of IL-6 by 68%.

With reference to muscle TNF concentrations (Fig 3B), no differences were observed between the experimental groups.

## Discussion

The aim of this study was to verify the effects of aerobic exercise associated with TRP supplementation on hyperalgesia, as well as on cortisol, IL-6 and TNF concentrations in 12-month-old female rats with experimental FM. It was found that by the end of 3 weeks of intervention LIAE alone reduced bilateral hyperalgesia (~ 151%) and serum cortisol concentrations (72%), while TRP supplementation itself diminished bilateral hyperalgesia (~ 57%) and cortisol concentrations (67%). Although, muscle TNF concentrations were not affected, the association of LIAE with TRP supplementation further reduced bilateral hyperalgesia (~ 162%), being an important decrease (68%) in muscle IL-6 concentrations observed, though the serum cortisol concentrations backed off to 45%.

The rat model used in the present study showed bilateral hind paw mechanical hyperalgesia that persisted over three weeks. We also observed elevated serum cortisol and muscle IL-6 concentrations in these animals over this period. There are evidences on that stress associated with high serum cortisol concentrations may exacerbate musculoskeletal pain in FM patients [34], and potentiate the pronociceptive effects of inflammatory cytokines such as IL-6 and TNF [8, 35]. Although the FM induction model used in this study is considered non-inflammatory [30], previous studies [35,36] on non-inflammatory stress models (i.e. sleep deprivation and sound stress) have observed that stress induces the persistent elevation of pro-inflammatory cytokines such as IL-6 and TNF. Despite that, in the present study no significant differences in muscle TNF concentrations between control and FM animals were observed. Considering that the influence of HPA axis activity and systemic hypercortisolemia on the pathophysiology of FM is controversial [5, 8, 37, 38, 39], which leads to the existence of a considerable heterogeneity of FM patients, it is possible that the elevated cortisol shown here in the model of repeated acidic saline is associated with only a sub-population of FM patients.

Concerning the effects of exercise, we observed that the LIAE program applied was efficient in returning bilateral mechanical hyperalgesia to control levels on the first week of the intervention, which persisted until the end of intervention (i.e. week 3). Such exercise benefit was followed by a reduction in serum cortisol concentrations. However, the observed reduction in muscle concentrations of IL-6 did not reach statistical significance. Musculoskeletal exercises release neurotransmitters, such as noradrenaline and serotonin, and activates specific receptors that helps to reduce stress-indicative scores [40, 41, 42]. Long-term aerobic activities increase the plasma concentrations of free tryptophan (TRP-F), whereas the concentrations of large neutral amino acid (LNAA) are reduced as a result of its increased uptake and oxidation by the exercised muscles [43]. Thus, the TRP-F/LNAA ratio decreased which augments the locomotion of TRP-F to the brain, thereby increasing the cerebral serotonin concentrations [44, 45]. Since serotonin is responsible for stimulating the HPA axis in response to stress [23], it is possible that our LIAE program has increased the concentrations of cerebral serotonin and thus reduced the release of cortisol in this model of FM. Such possibility warrants further investigations.

Regarding the results of TRP supplementation alone, it diminished bilateral hyperalgesia and serum cortisol concentrations to lesser extensions as compared to exercise. Tryptophan supplementation has been shown to improve the function of cerebral serotonin, which helps reducing the release of cortisol in stressful situations [24]. Such effect is thought to occur because the consumption of supplements rich in TRP increases their plasma proportion over the sum of the other LNAA giving TRP the advantage in the competition for accessing to the brain [22]. In this study, the TRP concentration in the control diet was 2.5g/kg while in the TRP supplemented it was 7.6g/kg. Thus, TRP/LNAA ratio in the TRP diet was approximately three times higher than that in the control diet (TRP/LNAA = 15.32 and TRP/LNAA = 5.4, respectively). In addition, weight gain and food consumption did not differ significantly between groups, ensuring that similar amounts of diet were consumed by the animals throughout the study period. Therefore, the higher TRP/LNAA ratio of the TRP supplemented diet appears to have favored the increase of brain TRP which would result in reduced serum cortisol and muscle IL-6 concentrations in the TRP supplemented group. Despite that, taking into consideration that tryptophan is a precursor of serotonin and is closely linked with psychiatric disorders, which consequently affects stress the absence of other behavioral tests (i.e. anxiety, stress) is a limitation of the present study.

More important, concerning the combination of treatments, we found that the association of TRP supplementation with LIAE did not further reduced bilateral hyperalgesia significantly (i.e. 11% vs exercise), being an important decrease in muscle IL-6 concentrations observed (~ 68%), though the serum cortisol concentrations backed down (i.e. 18% vs exercise). These results indicate that combination of treatments generated a synergy of the isolated effects on a biomarker indicative of inflammation, but not on that of stress and on hyperalgesia.

In this sense, the suggestion of whether is necessary or not adding TRP supplementation to LIAE in FM therapy warrants cost-benefit analyzes of the patient health conditions. On this wise, it has to be considered that little is known about the ideal amounts of TRP consumption. Although, TRP supplementation is related to improvements of symptoms such as mood, cognitive status and fatigue in patients undergoing chronic stress [20,21], its chronic and excessive consumption may be associated with increases in oxidative stress and in the risk of cardiovascular diseases [46,47]. Therefore, further studies on the safety of TRP supplementation in the management of FM patients are needed to determine accurate amounts and time of TRP consumption to achieve the benefits of supplementation. Moreover, since we observed that serum cortisol concentrations pulled back, behavioral assessments (i.e. fatigue, stress, anxiety and depression) should also be performed.

Finally, these results have clinical relevance as it gives insights into the potential of combining non-pharmacological therapies in the management of FM, specially it brings about the need for new researches to investigate the mechanisms involved in the behavioral and biochemical effects observed here.

In conclusion, the association of TRP supplementation to LIAE does not potentiate significantly the reduction of bilateral mechanical hyperalgesia promoted by LIAE in 12-month old female rats with experimental FM, however an important decrease in IL-6 is evident.

## Supporting information

S1 Table Concentrations of inflammatory cytokines and cortisol. (PDF)

S2 Table Mechanical hyperalgesia. Withdrawal threshold of right paw.
(PDF)

S3 Table Mechanical hyperalgesia. Withdrawal threshold of left paw.
(PDF)
