# Supplementary material for: Does aerobic exercise associated with tryptophan supplementation attenuates hyperalgesia and inflammation in female rats with experimental fibromyalgia?
Source: PLoS One. 2019 Feb 20;14(2):e0211824. doi: 10.1371/journal.pone.0211824 (PMC6382124; doi:10.1371/journal.pone.0211824)
Supplement: S1 Table — (PDF) [file pone.0211824.s001.pdf]

Groups	pg/mg IL-6	pg/mg TNF-α	ng/mL Cortisol
CON	11,91	0,21	13,233
CON	26,56	0,15	9,429
CON	26,9	0,15	13,961
CON	28,42	0,3	12,13
CON	22,29	0,22	19,589
CON	11,08	0,24	13,961
F	69,3	0,28	47,797
F	52,19	0,28	30,85
F	30,85	0,2	40,637
F	136,84	0,24	82,5
F	32,57	0,2	112,846
F	33,57	0,25	outlier
FE	56,68	0,2	16,766
FE	16,24	0,29	10,041
FE	48,59	0,17	23,116
FE	27,72	0,23	16,644
FE	37,47	0,2	16,191
FE	41,49	0,23	21,774
FES	13,24	0,13	17,209
FES	24,02	0,22	26,766
FES	22,18	0,33	40,755
FES	34,58	0,27	17,134
FES	16,01	0,23	48,08
FES	4,86	0,27	21,899
FS	40,32	0,2	34,336
FS	13,81	0,16	18,522
FS	22,28	0,15	22,66
FS	31,57	0,18	18,075
FS	24,58	0,1	14,148
FS	19,07	0,24	14,984
