# Supplementary material for: Does aerobic exercise associated with tryptophan supplementation attenuates hyperalgesia and inflammation in female rats with experimental fibromyalgia?
Source: PLoS One. 2019 Feb 20;14(2):e0211824. doi: 10.1371/journal.pone.0211824 (PMC6382124; doi:10.1371/journal.pone.0211824)
Supplement: S2 Table — Withdrawal threshold of right paw. (PDF) [file pone.0211824.s002.pdf]

Animals	Groups	Prein D	Posin D	Week 1D	Week 2D	Week 3D
1	CON	36,38	40	35,4	35,5	35,4
2	CON	36,315	35,1	33,86	34,5	33
3	CON	35,73	38,1	35	35,5	36,7
4	CON	36,56	38,1	35,5	36	36
5	CON	37,36	39,5	35,9	35,3	36
6	CON	38,065	31,1	35,83	36	37,1
7	CON	38,015	36,5	33,1	36,16	34,9
8	CON	37,98	35,86	35,76	36,3	35,7
1	F	35,86	19,4	14,7	15,8	14
2	F	36,395	16,63	14	14,1	15,3
3	F	35,71	15,93	14,3	13,4	13,3
4	F	36,616	14,1	12	13,6	14,75
5	F	36,8	22,23	12,3	13,76	12,83
6	F	35,78	18,6	14,9	14,2	15,1
7	F	36,58	15	14,2	11,93	13,8
8	F	36,35	11,6	14,8	14,4	15,7
1	FE	36,25	13,83	36,2	36,23	38,1
2	FE	36,5	12	37,8	36	34,9
3	FE	37,3	12	37,8	35,4	36,26
4	FE	35,45	12,23	36,9	35,23	35,75
5	FE	35,65	12,06	36,5	36	36
6	FE	36,95	12,1	35,4	35,9	36
7	FE	37,5	12,9	36,9	36	37,26
8	FE	36,2	12,1	36,3	35,8	36,25
1	FES	36,7	13,3	35,93	37,43	41,8
2	FES	36,2	10,73	37,36	37,1	39,2
3	FES	36,4	11	36,5	37,5	38,6
4	FES	36,4	12,2	35,16	37	38,1
5	FES	36,8	10,4	36,33	38,53	37,6
6	FES	35,7	12,9	35,6	37,26	37,93
7	FES	37,1	10,33	34,3	38,6	37,93
8	FES	36	13	36,6	37,16	36
1	FS	36,6	14,5	21,2	18,16	16,3
2	FS	37,6	12,13	12,16	24,66	26,12
3	FS	36,7	13,15	19,7	23	24,16
4	FS	36,4	14,5	17,26	16	21
5	FS	36,4	12,93	16,5	19,53	24,3
6	FS	37	12,43	23,3	19,83	23
7	FS	35,8	12,56	17,5	21,33	26,5
8	FS	36,9	13,16	18,56	24,15	25,5
