# Supplementary material for: Does aerobic exercise associated with tryptophan supplementation attenuates hyperalgesia and inflammation in female rats with experimental fibromyalgia?
Source: PLoS One. 2019 Feb 20;14(2):e0211824. doi: 10.1371/journal.pone.0211824 (PMC6382124; doi:10.1371/journal.pone.0211824)
Supplement: S3 Table — Withdrawal threshold of left paw. (PDF) [file pone.0211824.s003.pdf]

Animals	Groups	Prein E	Posin E	Week 1E	Week 2E	Week 3E
1	CON	37,3	38	35,4	36,3	35,9
2	CON	36,86	36,7	35,5	35,8	34,8
3	CON	36,45	37,9	34,6	35,66	34,5
4	CON	35,415	37,83	35,36	36,66	37,5
5	CON	36,545	36,6	35,4	37,66	37,9
6	CON	37,565	31,6	35,16	36	36
7	CON	37,465	34,7	36,1	33,33	36,6
8	CON	37,38	36,5	36,1	35,4	36,5
1	F	34,93	14,96	14,3	11,8	18,8
2	F	35,68	17,1	14	15	13
3	F	37,08	17,5	13,8	16,1	15,5
4	F	35,2	16,3	15,6	12,63	13,9
5	F	36,095	18,4	12,26	18,2	13,6
6	F	36,7	18,6	16,2	13,16	14,7
7	F	36,495	13	15,75	12,3	16,76
8	F	37,48	13,8	14,65	14,6	12
1	FE	36,5	12,7	38	34,7	37,36
2	FE	36,95	12,8	38,8	36	36,4
3	FE	37,55	12	38,1	36,23	37
4	FE	37,7	14,7	38,4	35,2	34,6
5	FE	36,9	12,5	38,2	36	36,96
6	FE	36,95	15,8	36,5	35,23	36,93
7	FE	38,3	14,15	35,8	36,8	37,2
8	FE	36,4	13,6	36,8	34,8	36,25
1	FES	35,5	10,6	36,66	36,9	39,7
2	FES	36,2	12,43	36,5	36,16	35,3
3	FES	36,7	11	36,83	39,3	40,5
4	FES	36,4	10	36,93	37,9	37,76
5	FES	36,8	12,83	37,16	38,2	38,05
6	FES	36,3	13	37,66	36,83	37,6
7	FES	36,7	11,86	36	36	39
8	FES	35,7	10,36	37	36,6	37,86
1	FS	37	10,3	20,76	16	16,3
2	FS	37	13,9	12,76	27	25,9
3	FS	36,4	11	21,5	15,5	22
4	FS	37	14,16	16,26	23,26	20,13
5	FS	37	13,16	16,33	20,15	21
6	FS	37,5	14,25	23,3	19,66	26
7	FS	37,9	13,93	18,83	22,5	25,8
8	FS	36,9	14,5	17,5	24,75	23,2
